# Improving sleep in a population at high risk of trauma: A pilot study examining self-reported sleep, psychological symptomology and actigraphy measured night-time sleep

**DOI:** 10.1192/j.eurpsy.2022.2094

**Published:** 2022-09-01

**Authors:** D. Maguire, C. Armour, S. Lagdon, M. Ruddock, T. Moore, M. Milanak

**Affiliations:** 1 Queen’s University Belfast, School Of Psychology, Belfast, United Kingdom; 2 Queen’s University Belfast, Psychology, Belfast, United Kingdom; 3 Ulster University, Psychology, Coleraine, United Kingdom; 4 Randox Health, Bioscience, Belfast, United Kingdom; 5 Medical University of South Carolina, Psychiatry & Behavioral Sciences, Charleston, United States of America

**Keywords:** Trauma, PTSD, sleep, actigraphy

## Abstract

**Introduction:**

Sleep disturbances (SDs), such as insomnia or regular nightmares, are associated with multiple mental health disorders, most notably PTSD, where SDs are reported in up to 92% of cases. Examining the effect of changing sleep on psychological symptomology is essential to develop the evidence base on the contribution of sleep to mental resilience.

**Objectives:**

To examine the effect a short skills-based sleep intervention on psychological symptomology and actigraphy measured sleep.

**Methods:**

A 4-session sleep skills training programme was used to treat active SDs in participants likely to have experienced occupation-associated trauma, namely military and first responders.

**Results:**

Nineteen participants were included in the study. Insomnia Severity Index (ISI) measured; difficulty sleeping, difficulty staying asleep, waking too early, sleep satisfaction, sleep interference on quality of life and total ISI insomnia score improved significantly post-treatment (M = 9.44, SE = 7.35, p <0.001). No significant difference was identified post-treatment for actigraphy-measured sleep. The severity of depression (M = 5.27, SE = 1.41, p = 0.002), anxiety (M = 5.07, SE = 1.66, p = 0.008), and PTSD symptoms among participants with likely PTSD, were significantly lower following treatment (M = 29.4, SE = 4.19, p = 0.002).

**Conclusions:**

A short sleep skills intervention based on CBT-I was effective at reducing self-report insomnia symptoms and severity of psychological symptomology but failed to improve actigraphy sleep metrics. These findings highlight a differing contribution of night-time sleep and current insomnia symptoms to the severity of self-reported psychological symptomology.

**Disclosure:**

No significant relationships.

